# Biomonitoring Environmental Exposure in Syrian Refugees in Lebanon

**DOI:** 10.3390/epidemiologia5020021

**Published:** 2024-06-19

**Authors:** Malek Alaouie, Gera M. Troisi

**Affiliations:** Institute for Health, Medicine and Environments, Brunel University London, Uxbridge UB8 3PH, UK

**Keywords:** biomonitoring, environmental epidemiology, refugees, respiratory health

## Abstract

Over one million Syrian refugees have been residing in substandard living conditions in Lebanon for the past decade. Non-invasive biomonitoring of fractional exhaled nitric oxide (FeNO) as a pulmonary inflammation biomarker was conducted following and preceding indoor environmental assessments (which revealed elevated mould counts in informal tented settlements and non-residential shelters) to further evaluate effects of environmental exposure to indoor contaminants. Results of biomonitoring (*n* = 57) provided some insight regarding existing respiratory conditions and the possible implementation of minimally invasive methods to establish susceptibility profiles in Syrian refugees amid limited access to healthcare. The clinical interpretation of FeNO results suggested possible persistent exposure to allergens in addition to significant type 2 inflammation in some subjects. These findings warrant the need to expand this study, investigate other biomarkers, and attempt to correlate findings with environmental conditions to evaluate if a dose–response relationship exists.

## 1. Introduction

Lebanon has witnessed a massive influx of Syrian refugees since 2010, which was met with various challenges mainly due to the lack of preparedness of the hosting country, which had been suffering from an ongoing economic and political crisis for several years. Lebanon became the second largest Syrian refugee hosting country, after Turkey, in 2017, with over one million refugees registered with the United Nations High Commissioner for Refugees (UNHCR) [1,2,3]. Compared to the Lebanese host population, Syrian refugees required more medical care, whereby around 60% of medical care needs of children were attributed to respiratory problems and the majority of medical care needs of adults were reported as infections and communicable diseases [4]. Access to healthcare and secondary care has been challenging for Syrian refugees mainly due to socio-economic factors and a competing host community, of which 50% are uninsured and sponsored by the Ministry of Public Health [4,5,6,7]. In 2015, the most prevalent reported chronic condition among refugees under 17 was chronic respiratory diseases, including asthma, emphysema, chronic bronchitis, and chronic obstructive pulmonary disease, accounting for 12.9% of reasons for hospitalization. Asthma and pulmonary disease, which were among the five most prevalent chronic conditions reported by surveyed Syrian refugees in 2015, grew to 19% in 2021, becoming the highest reported chronic conditions among the total refugee population [4,8]. Counting as more than 50% of the Syrian refugee population in Lebanon, children with asthma and respiratory diseases are at even higher risk in terms of susceptibility to indoor pollutants [9,10].

A preceding study conducted in the four major governorates of Lebanon revealed several significant associations between categories of shelter and mould concentrations, and further established strong associations between certain mould types and shelter conditions [11]. The reported medical conditions of Syrian refugees have not been correlated with housing, shelter type, nor environmental factors. Moreover, as access to medical information was challenging due to confidentiality and lack of formal diagnosis, this research was designed to obtain cross-sectional epidemiological data that are both minimally invasive and prompt by collecting fractional exhaled nitric oxide (FeNO) samples with the aim to create a respiratory health and susceptibility profile for refugees based on shelter type without attributing health conditions to housing or shelter type, as many refugees arrived in Lebanon with pre-existing medical conditions. Thus, it is of utmost importance to investigate emerging health effects in refugee settlements in Lebanon, especially for younger age groups, due to the fact that newborn children and children up to the age of about 14 years of refugee families who fled the war in 2010 were born in refugee camps.

In epidemiological studies, biomonitoring is used in combination with health data to address the biological or toxic effects of pollutants, also known as the body burden. This tool can be an indicator of temporal exposure trends in relation to geographic characteristics and identifying vulnerable subpopulations [12]. Although biomonitoring cannot indicate the source of exposure, it can document routes of exposure such as inhalation, dermal absorption, and ingestion. Most importantly, biomonitoring helps establish or rule out correlations between environmental exposure and associated health effects [13].

Biomonitoring includes sub-organismal measurements known as biomarkers, which are defined as biochemical responses and chemically induced histopathological alterations [14]. There are three types of biomarkers: markers of exposure, markers of effect, and markers of susceptibility [15]. Biomarkers of exposure characterize tissue and body fluids chemical residues, in addition to metabolites of xenobiotic compounds and exposure-related physiological changes. Biomarkers of effects are quantifiable biochemical and physiologic changes resulting from exposure ranging from biomolecular changes at the sub-cellular level to organ and tissue level changes. Biomarkers of susceptibility, such as polymorphisms of xenobiotic compounds, reflect fundamental characteristics of organisms that render them prone to adverse effects of exposure to specific substances [12,15].

Lam and Gray (2003) summarized the benefits of adopting biomarkers in environmental assessments. The authors argued that biomarkers are effective tools as early warning signals of adverse biological effects when they are optimally sensitive. Furthermore, the advantage of biomarkers over chemistry-based surveillance is in the ability of biomarkers to indicate biological effects. Biomarkers are also effective in reflecting the overall toxicities of complex mixtures. In this regard, specific biomarkers indicate that toxicity occurs when chemicals bind to specific receptors to trigger a toxic action. These responses are developed into bioassays, such as an enzyme-linked immunosorbent assay (ELISA), which are considered economical compared to the chemical analysis of toxins [16]. The specificity and sensitivity of biomarkers are extremely important in justifying their use and receiving accurate exposure or effect information.

Moreover, blood, serum, and plasma are the biological matrices generally used, as they have well established standard operating procedures for sampling. Urine and other matrices are also used based on the type of toxin or pollutant. The choice of matrix also depends on whether it is invasive or non-invasive [12].

Infection, trauma, or exposure to exogenous toxins and irritants stimulates reactions such as inflammation and oxidative stress. In order to detect and monitor cytokine-mediated inflammation and oxidant stress, exhaled gases provide an effective tool of measurement [17]. Human breath contains endogenous compounds, such as inorganic gases (NO and CO), VOCs, and non-volatile substances such as isoprostanes, peroxynitrite, and cytokines [18]. These endogenous compounds have been vastly investigated in the literature for their diagnostic potential [19,20].

NO is produced by different types of pulmonary cells, including inflammatory, endothelial, and airway epithelial cells, and has been implicated in the pathophysiology of lung disease, playing key roles in vasodilation and bronchodilation, and as an inflammatory mediator [17,21]. Measurement of exhaled NO has been suggested for non-invasive diagnosis and monitoring of diseases in which airway excretion of NO is altered [22]. Additionally, the fraction of NO in exhaled air, also known as FeNO, is highly correlated with eosinophilic airway inflammation, and its measurement has been adopted in the diagnosis of respiratory illnesses such as asthma, COPD, cystic fibrosis, and primary ciliary dyskinesia [22,23]. The presence of NO in the airways is due to the activation of the transcription factor NK-kB by cytokines in epithelial cells, which triggers the production of the enzyme inducible nitric oxide synthetase (iNOS), which is responsible for the production of NO [24].

Tang et al. (2017) collected exhaled breath condensate (EBC) and serum from 102 acute respiratory distress syndrome (ARDS) ICU patients aged between 42 and 73, before treatment. EBC and serum NO from ARDS patients were significantly higher than those from controls (47.81 µmol/L EBC and 48.45 µmol/L serum NO compared to 15.65 µmol/L and 18.76 µmol/L, respectively, from controls). Although levels were significantly lower 5 days after treatment was administered, EBC and serum NO were still higher in treated ARDS patients than controls’ levels were. These findings suggest that quantifying EBC NO levels can help in the evaluation of treatment efficacy and determining prognosis of ARDS [25].

A study conducted by Nguyen-Thi-Bich et al. in 2016 attempted to evaluate correlations between FeNO and atopic status, blood eosinophil levels, FCER2 mutation, and asthma control in 42 Vietnamese children with uncontrolled asthma. FeNO levels were significantly higher in patients with a positive skin prick test for respiratory allergens (*p* < 0.05) and significantly correlated with blood eosinophil levels (*r* = 0.5217; *p* = 0.0004), inferring that FeNO level is a feasible biomarker for the prediction of the clinical and biological status of asthmatic children [26]. Another study of asthmatic children, performed by Brzozowska et al. (2015), aimed to show correlations between FeNO levels and cytokine concentrations. Their results revealed a significant positive correlation between the FeNO level and IL-2, monocyte hemoattractant protein-1 (MCP-1), platelet-derived growth factor BB (PDGFBB), and tissue inhibitory of metalloproteinase 2 (TIMP2), and suggested that EBC may be a useful non-invasive tool to phenotype asthma [27].

Further interpretation of FeNO results could also suggest the likelihood of type 2 (T2) inflammation, which is used in the aetiology and pathogenesis of asthma and is driven by the production of pro-inflammatory type 2 cytokines [28,29,30,31]. Intermediate levels suggest the likelihood of T2 inflammation, while high levels suggest significant T2 inflammation [32]. No body of evidence exists to conclude correlations between FeNO and severe asthma; nevertheless, results from clinical studies and research do correlate with the management of asthma and the maintenance of inhaled corticosteroids [32,33,34,35].

## 2. Materials and Methods

A NIOX VERO^®^ (Circassia AB, Uppsala, Sweden) airway inflammation monitor was used to measure FeNO from Syrian refugees who were registered in the Save the Children beneficiary database, following ethical approval obtained from the Brunel Research Ethics Committee. The portable device includes a breathing handle connected to a display instrument that includes an NO sensor. Monitoring occurred inside refugee shelters, and subjects were instructed to exhale forcefully and steadily into the breathing handle through a disposable patient filter and mouthpiece attached to the handle. The device displayed animation especially to guide children on how to maintain a steady flow. The device allowed measurement of exhaled breath via a 10 or 6 s option, depending on the age of the subject [36].

The original study design aimed to monitor refugees in the same Lebanese provinces and shelter types covered in the preceding study [11]. A sample size representing the refugee population registered with UNHCR was calculated to be 385, with a 95% confidence interval and a 5% error margin. Other nationalities that did not share the same humanitarian and socio-political profile of Syrian refugees were excluded from this study. Additionally, individuals with recently diagnosed infections or exhibiting infectious symptoms were also excluded from this study. This study was limited to 2 days due to national security concerns following the aggravation of the civil uprising in Lebanon, which began in October 2019, and hindered transportation to refugee settlements, with restricted mobilization of NGOs, limiting the sample size to 57. This study was then discontinued following the COVID-19 pandemic due to shelter-in-place orders and the nature of the study, which was considered an aerosol-generating procedure (AGP) in a non-controlled environment.

Subjects were asked whether they were diagnosed with respiratory illnesses and whether they were smokers, then further labelled by age group (child “C”, adult “A”, or elderly “E”) and gender (male “M” or female “F”) (Table 1). The 57 selected subjects, of whom 60% were children, delivered a single exhaled breath measurement each. FeNO measurements were taken between December 2019 and January 2020 in non-residential and non-permanent shelters only, in the South governorate and Bekaa regions of Lebanon, respectively, with the following demographic distribution.

## 3. Results and Discussion

FeNO results were interpreted as low, intermediate, or high, based on the official clinical practice guidelines of the American Thoracic Society (ATS) and the National Institute for Health and Care Excellence (NICE). The ATS cut off level for high levels of FeNO is 50 parts per billion (ppb) compared the 40 ppb set by the NICE guidelines, which were adopted for this study’s clinical interpretation. Accordingly, the NICE intermediate levels were 25–40 ppb and 20–35 ppb for adults and children, respectively [35,37].

### 3.1. Non-Residential Shelters

**Figure 1:**
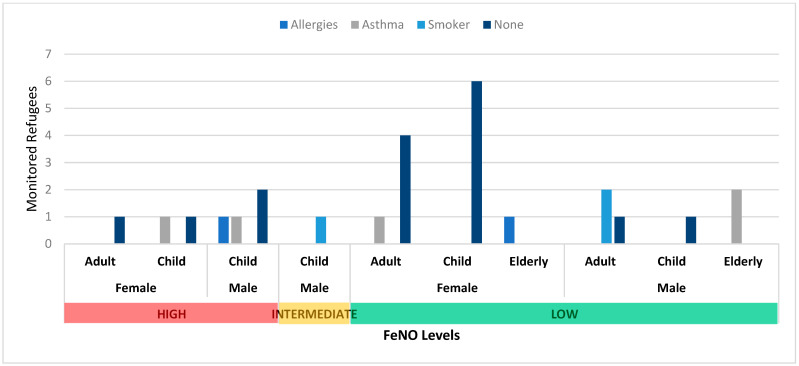
Non-residential FeNO results.

Of the 26 monitored refugees in non-residential shelters, six children (four M and two F) reported high levels of FeNO (>40 ppb), two of whom reported asthma and one of whom reported an allergy. Only one adult (F) had high levels of FeNO, and one child (17 years, M), who was a smoker, had intermediate levels (Figure 1).

### 3.2. Non-Permanent Shelters

**Figure 2:**
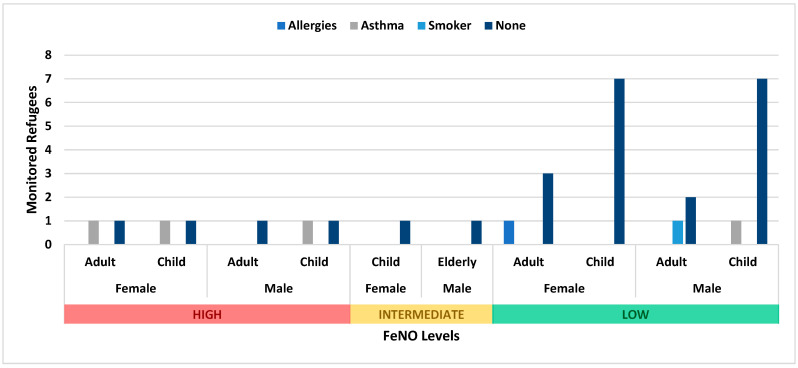
Non-permanent FeNO results.

Monitoring results from non-permanent shelters reported high FeNO levels in four children (two M and two F), two of whom had asthma. Furthermore, three adults (two F and one M) reported high levels of FeNO, whereas intermediate levels were reported in one child (F) and one elderly person (M) (Figure 2).

**Figure 3:**
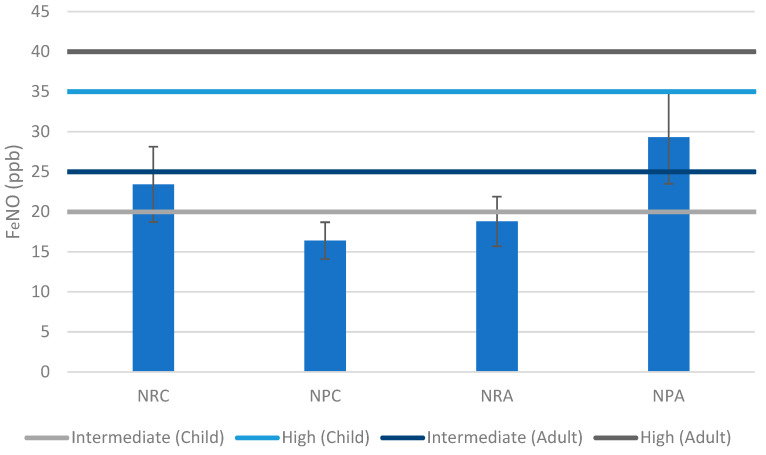
FeNO levels, means, and standard errors. NRC (non-residential children); NPC (non-permanent children); NRA (non-residential adults); NPA (non-permanent adults).

The mean FeNO level for children residing in non-residential shelters was 23.4 ± 4.7 ppb compared to 16.4 ± 2.3 ppb for those in non-permanent shelters. As for adults, the mean FeNO level was 18.8 ± 3.1 ppb in non-residential shelters compared to 29.3 ± 5.8 ppb in non-permanent shelters (Figure 3).

The mean FeNO level exceeded the intermediate category for children in non-residential shelters and for adults in non-permanent shelters. The difference in the children’s age group results could be due to the fact that children residing in non-residential shelters were observed to be spending more time indoors due to the limitation of outdoor activities in such shelters, which included repurposed classrooms. On the other hand, children of non-permanent shelters, which are informal tented settlements established on or near agriculture fields, were observed to be spending more time outdoors. Furthermore, the preceding study revealed that non-residential shelters had the highest mean mould concentration, followed by non-permanent shelters [11]. As for the adult age group, the number of monitored adult females in non-residential shelters was twice that of adult males. Some studies suggested that older males had higher median FeNO levels than females, and others found a negative correlation with female age [38,39,40,41]. Nevertheless, although mean levels may indicate a higher susceptibility of children in non-residential shelters and adults in non-permanent shelters, the small sample size and unaccounted-for environmental factors and baseline conditions could not substantiate this conclusion. Further research is required that takes confounding factors into consideration. Although no significant correlation was observed between the age of subjects and FeNO levels (R^2^ = 0.7%) in both types of shelters, this may have been due to the small sample size.

Table 2 below highlights interpretations of FeNO levels reported by symptomatic and asymptomatic monitored refugees in both types of shelters.

**Figure 4:**
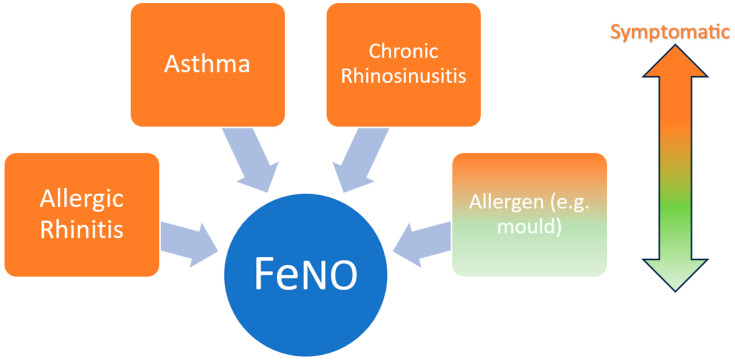
Environmental and underlying medical conditions leading to the production of FeNO in symptomatic and asymptomatic subjects.

The majority of monitored refugees were asymptomatic, as highlighted in Table 2. One of the clinical management guidelines for asymptomatic patients with high and intermediate levels of FeNO is to consider high baseline NO production and/or persistent allergen exposure potentially due to subclinical inflammation of lower airways with the absence of symptoms [32,42,43]. Additional confounding factors, such as gender, age, smoking, nutrition, cirrhosis, viruses, and bacterial infections, should also be taken into account when interpreting FeNO results [44,45,46]. Although other shelter types, such as residential shelters, were not assessed, as mentioned, the previous study revealed that non-residential shelters had the highest concentrations of mould, followed by non-permanent shelters [11]. Repetitive long-term exposure to mould and other aeroallergens in these shelter categories could explain the high FeNO levels observed in asymptomatic subjects [47]. Figure 4 depicts the underlying and environmental conditions that lead to the production of FeNO.

## 4. Conclusions, Limitations, and Future Research

The findings of this study, although limited, clearly suggest an urgent need to repeat and broaden this study to include residential shelters and an increased sample size to investigate the presence of any significant correlation between FeNO levels and environmental conditions in shelters. Furthermore, a more inclusive study would benefit refugee health management by establishing a health susceptibility profile of refugees in circumstances where continuous clinical management cannot be maintained. As previous surveys reported an increasing rate of hospitalization due to asthma and pulmonary diseases, this type of data will assist NGOs in the proper deployment of resources and households, and can also be used to communicate concerns to policy makers and reduce the rate of hospitalization and additional burden on the host country’s health system by acting as an early warning system.

Although several environmental factors are impacting refugee wellbeing, further epidemiological studies are needed to determine the quantitative impact (dose–response relationship) of these factors on the health of refugees. Stakeholder meetings, including clinicians, indoor air quality specialists, public specialists, and humanitarian aid and government agencies, should be urgently convened to determine whether indoor air quality is significantly influencing the health and wellbeing of this population. Although FeNO detection holds potential benefits as a non-invasive biomonitoring method, it does have limitations due to previously mentioned confounding factors. Of particular value would be exploring other biomarkers and expanding the biomonitoring capability and application of portable electrochemical sensors and non-invasive methods to facilitate the greater sample size required for statistical robustness in a cost-effective and time efficient manner, while minimising adverse impact on study subjects, particularly the elderly and children.

## Figures and Tables

**Table 1:** FeNO population data (*n* = 57).

Type of Shelter	Gender	Children (<18)	Reported Conditions	Adults (18 < 60)	Reported Conditions	Elderly (>60)	Reported Conditions
Non-residential	F	8	Asthma (1)				
				6	Asthma (1)	1	Allergies (1)
			
	M	6	Smoker (1)				
			Asthma (1) Allergies (1)	3	Smoker (2)	2	Asthma (2)
Non-permanent	F	10	Asthma (1)	6	Asthma (1) Allergies (1)	0	None
	M	10	Asthma (2)	4	Smoker (2)	1	None

**Table 2:** Clinical interpretation of FeNO levels in symptomatic and asymptomatic refugees.

FeNO Levels	T2 Inflammation	Symptomatic	Asymptomatic	Reference
Low	Unlikely	6	34	
Intermediate	Likely	0	3	[32]
High	Significant	6	8	

## Data Availability

Data underpinning this publication can be accessed from Brunel University London’s data repository, Figshare, here under a CCBY license: https://doi.org/10.6084/m9.figshare.25360525.

## References

[1] Lambert H. (2017). Temporary refuge from war: Customary international law and the Syrian conflict. Int. Comp. Law Q..

[2] UNHCR (2017). 3RP Regional Refugee & Resilience Plan 2017–2018.

[3] UNHCR Refugee Statistics. https://www.unhcr.org/refugee-statistics/download/?url=2bxU2f.

[4] John Hopkins Bloomberg School of Public Health, Mèdecins Du Monde, International Medical Corps, American University of Beirut, UNHCR (2015). Humanitarian Aid and Civil Protection Syrian refugee and Affected Host Population Health Access Survey in Lebanon. https://data.unhcr.org/en/documents/details/44869.

[5] Blanchet K., Fouad F.M., Pherali T. (2016). Syrian refugees in Lebanon: The search for universal health coverage. Confl. Health.

[6] El-Khatib Z., Scales D., Vearey J., Forsberg B.C. (2013). Syrian refugees, between rocky crisis in Syria and hard inaccessibility to healthcare services in Lebanon and Jordan. Confl. Health.

[7] Amnesty International (2014). Agonizing Choices: Syrian Refugees in Need of Health Care in Lebanon.

[8] UNHCR (2022). Health Access and Utilization Survey among Syrian Refugees in Lebanon.

[9] Li Y., Feng L., Chen B., Kim H., Yi S., Guo Y.L., Wu C. (2016). Association of urban particle numbers and sources with lung function among children with asthma or allergies. Sci. Total Environ..

[10] Lin L., Tsai M., Chen M., Ng S., Hsieh C., Lin C., Lu F.L., Hsieh W., Chen P. (2018). Childhood exposure to phthalates and pulmonary function. Sci. Total Environ..

[11] Alaouie M., Troisi G.M., Saliba N., Shaib H., Hajj R., El Hajj R., Malak S., Jakarian C., Jaafar W. (2023). Fungal Exposure and Shelter Assessment in Syrian Refugee Settlements in Lebanon. Aerobiology.

[12] WHO (2015). Regional Office for Europe Human Biomonitoring: Facts and Figures.

[13] Sexton K., Needham L.L., Pirkle J.L. (2004). Human Biomonitoring of Environmental Chemicals.

[14] Hanson N., Halling M., Norin H. (2013). Biomarkers for Environmental Monitoring Suggestions for Norwegian Monitoring Programs.

[15] National Research Council (1987). Biological Markers in Environmental Health Research.

[16] Lam P.K.S., Gray J.S. (2003). The use of biomarkers in environmental monitoring programmes. Mar. Pollut. Bull..

[17] Paredi P., Kharitonov S.A., Barnes P.J. (2002). Analysis of Expired Air for Oxidation Products. Am. J. Respir. Crit. Care Med..

[18] US Consumer Product Safety Commission (1996). Indoor Air Pollution: An Introduction for Health Professionals.

[19] Horvath I., Loukides S., Wodehouse T., Kharitonov S.A., Cole P.J., Barnes P.J. (1998). Increased levels of exhaled carbon monoxide in bronchiectasis: A new marker of oxidative stress. Thorax.

[20] Uasuf C.G., Jatakanon A., James A., Kharitonov S.A., Wilson N.M., Barnes P.J. (1999). Exhaled carbon monoxide in childhood asthma. J. Pediatr..

[21] Nathan C., Xie Q. (1994). Nitric oxide synthases: Roles, tolls, and controls. Cell.

[22] Taylor D.R., Pijnenburg M.W., Smith A.D., Jongste J.C.D. (2006). Exhaled nitric oxide measurements: Clinical application and interpretation. Thorax.

[23] Kelekci S., Sen V., Yolbas I., Uluca Ü., Tan I., Gürkan M.F. (2013). FeNO levels in children with asthma and other diseases of the lung. Eur. Rev. Med. Pharmacol. Sci..

[24] Pignatti P., Visca D., Loukides S., Märtson A., Alffenaar J.C., Migliori G.B., Spanevello A. (2022). A snapshot of exhaled nitric oxide and asthma characteristics: Experience from high to low income countries. Pulmonology.

[25] Tang K., Shao X., Liu F., Zhu B., Dong Z., Xu W., Yang Q. (2017). Correlation between nitric oxide content in exhaled breath condensate and the severity of acute respiratory distress syndrome. Int. J. Clin. Exp. Pathol..

[26] Nguyen-Thi-Bich H., Duong-Thi-Ly H., Thom V.T., Pham-Thi-Hong N., Dinh L.D., Le-Thi-Minh H., Craig T.J., Duong-Quy S. (2016). Study of the correlations between fractional exhaled nitric oxide in exhaled breath and atopic status, blood eosinophils, FCER2 mutation, and asthma control in Vietnamese children. J. Asthma Allergy.

[27] Brzozowska A., Majak P., Jerzyńska J., Smejda K., Bobrowska-Korzeniowska M., Stelmach W., Koczkowska M., Stelmach I. (2015). Exhaled nitric oxide correlates with IL-2, MCP-1, PDGF-BB and TIMP-2 in exhaled breath condensate of children with refractory asthma. Adv. Dermatol. Allergol./Postępy Dermatol. Alergol..

[28] Fahy J.V. (2015). Type 2 inflammation in asthma—Present in most, absent in many. Nat. Rev. Immunol..

[29] Dunican E.M., Fahy J.V. (2015). The role of type 2 inflammation in the pathogenesis of asthma exacerbations. Ann. Am. Thorac. Soc..

[30] Busse W.W., Kraft M., Rabe K.F., Deniz Y., Rowe P.J., Ruddy M., Castro M. (2021). Understanding the key issues in the treatment of uncontrolled persistent asthma with type 2 inflammation. Eur. Respir. J..

[31] Kosoy I., Lew E., Ledanois O., Derrickson W. (2022). Characterization of uncontrolled, severe asthma patients with type 2 inflammation (T2): Results from a physician survey across countries from Latin American, Eurasian Middle East regions and China. J. Asthma.

[32] CIRCASSIA (2020). Clinical Guidelines for The Interpretation of FeNO Levels. https://www.niox.com/en-us/feno-asthma/interpreting-feno/.

[33] Centre of Excellence in Severe Asthma (2019). Inflammation Biomarkers in the Assessment and Management of Severe Asthma—Tools and Interpretation. https://www.severeasthma.org.au/biomarkers-recommendation/.

[34] Chiappori A., De Ferrari L., Folli C., Mauri P., Riccio A.M., Canonica G.W. (2015). Biomarkers and severe asthma: A critical appraisal. Clin. Mol. Allergy.

[35] Dweik R.A., Boggs P.B., Erzurum S.C., Irvin C.G., Leigh M.W., Lundberg J.O., Olin A., Plummer A.L., Taylor D.R. (2011). An Official ATS Clinical Practice Guideline: Interpretation of Exhaled Nitric Oxide Levels (FeNO) for Clinical Applications. Am. J. Respir. Crit. Care Med..

[36] CIRCASSIA (2016). NIOX VERO Airway Inflammation Monitor.

[37] National Institute for Health and Care Excellence (2017). Asthma: Diagnosis, Monitoring and Chronic Asthma Management.

[38] Kumar R., Gupta N., Goel N. (2013). Correlation of atopy and FeNO in allergic rhinitis: An Indian study. Indian J. Chest Dis. Allied Sci..

[39] Czubaj-Kowal M., Nowicki G.J., Kurzawa R., Polak M., Ślusarska B. (2022). Factors Influencing the Concentration of Exhaled Nitric Oxide (FeNO) in School Children Aged 8–9-Years-Old in Krakow, with High FeNO Values ≥ 20 ppb. Medicina.

[40] Murugesan N., Saxena D., Dileep A., Adrish M., Hanania N.A. (2023). Update on the role of FeNO in asthma management. Diagnostics.

[41] Serbina N.V., Salazar-Mather T.P., Biron C.A., Kuziel W.A., Pamer E.G. (2003). TNF/iNOS-producing dendritic cells mediate innate immune defense against bacterial infection. Immunity.

[42] Such J., Francés R., Pérez-Mateo M. (2002). Nitric oxide in patients with cirrhosis and bacterial infections. Metab. Brain Dis..

[43] Zhang X., Xu Z., Lin J., Xie G., Lv C., Zhang M. (2023). Sex differences of small airway function and fractional exhaled nitric oxide in patients with mild asthma. Ann. Allergy Asthma Immunol..

[44] Olivieri M., Corradi M., Malerba M. (2007). Gender and exhaled nitric oxide. CHEST J..

[45] Janahi I., Saadoon A., Tuffaha A., Panneerselvam B. (2012). Effects of age, gender, and environmental exposures on exhaled nitric oxide level in healthy 12 to 18 years Qatari children. Ann. Thorac. Med..

[46] Zhang H., Shu L., Cai X., Wang Z., Jiao X., Liu F., Hou P., Wang L., Shan L., Chen N. (2013). Gender and age affect the levels of exhaled nitric oxide in healthy children. Exp. Ther. Med..

[47] Escamilla-Gil J.M., Fernandez-Nieto M., Acevedo N. (2022). Understanding the cellular sources of the fractional exhaled nitric oxide (FeNO) and its role as a biomarker of type 2 inflammation in asthma. BioMed Res. Int..

